# Dr. Luke P. Blackburn

**Published:** 1878-12-20

**Authors:** 


DR. LUKE P. BLACKBURN, of Ky., who will be remembered for his philanthropic efforts in behalf of the Yellow Fever Sufferers, has been announced as a candidate for Governor of Kentucky, and, it is believed, will be elected.

